# Evaluating the Hospital Learning Environment for Medical Interns in Uganda: a Crossectional Study at Three National Referral Hospitals

**DOI:** 10.21203/rs.3.rs-5675020/v1

**Published:** 2025-03-31

**Authors:** Musa Kirya, Aloysius G. Mubuuke, Ian Munabi, Charity Mutesi, Sarah Kiguli, Paul Matovu

## Abstract

**Background** Medical internship is a crucial phase where theoretical knowledge is translated into practical skills through supervised patient care. Globally, this training is grounded in work-based learning, but balancing educational objectives with service delivery often creates challenges. In developed settings, learning environments are integral to accreditation standards, while in Uganda, issues such as poor supervision, inadequate resources, and dissatisfaction among interns remain prevalent. Structural changes to internship rotations and frequent strikes further highlight the need for systematic evaluation. Despite these challenges, no formal feedback mechanisms exist to capture intern doctors' perspectives on their clinical learning environments. We sought to evaluate intern doctors' perceptions of their hospital learning environment and compare these perceptions across clinical departments in three National Referral Hospitals. **Methods** We conducted a cross-sectional study at Mulago, Kawempe, and Kiruddu National Referral Hospitals, enrolling 200 medical interns. Interns' perceptions were assessed using the Postgraduate Hospital Education Environment Measure (PHEEM) questionnaire. Institutional Review Board approval was obtained, and all participants provided informed consent. **Results** The meanlearning environment score for the three hospitals was 93.27 (maximum 160), with subscale scores of 31.35 (maximum 56) for autonomy, 37.37 (maximum 60) for teaching, and 23.65 (maximum 44) for social support. Analysis of variance of the mean scores revealed significant differences in perception of the learning environment regarding clinical rotation with the medical disciplines scoring higher than the surgical disciplines: Pediatrics ( p = 0.015) Internal medicine (p=0.05) compared to Obstetrics/Gynecology and Surgery. However, there were no significant differences between internal medicine and pediatrics or between surgery and obstetrics/gynecology. **Conclusion** The hospital learning environment across the three hospitals was rated as more positive than negative, though deficiencies were noted, particularly in social support and role autonomy. Medical disciplines of: Internal medicine and Pediatrics had better-rated learning environments than the surgical disciplines of: Surgery and Obstetrics and Gynecology. Clinical trial number: Not applicable.

Clinical trial number: Not applicable.

## BACKGROUND

The medical internship serves as a critical bridge in transitioning medical students to practicing physicians. It is a pivotal period during which theoretical knowledge is transformed into practical skills through supervised patient care(1).

Globally, medical internship training is grounded in a work-based learning model, where junior doctors gain knowledge and experience while providing clinical services (2). However, this dual role of education and service often creates tension between learning objectives and service delivery demands (3–5). To optimize this balance, deliberate efforts must be directed toward fostering a learning environment that is conducive to both education and clinical care. To achieve this balance, educators ought to develop a thought-through and organized learning structure that stipulates the interns' learning goals and outcomes. Largely this protects trainees from exploitative and unfavorable learning environments(6). This is so critical in the context of low-resource settings like Uganda because the severe deficiency of human resources for health tends to impose a view of an intern doctor as a service provider rather than a trainee(7). Such environments should be regularly and systematically assessed to ensure they meet the expectations of intern doctors and align clinical service with educational goals (8, 9). In the more developed settings, the internship learning environment constitutes one of the pillars of accreditation standards of the training program as opposed to low and middle-income countries like Uganda(10)

In Uganda, challenges within the internship program are evident, ranging from poor supervision and inadequate resources to dissatisfaction among interns about the training environment(11). In fact, on a global stage, several sources highlight the very significant role of organizational cultural contexts, overwhelming workload, and faculty engagement as key determinants of postgraduate trainee satisfaction in workplace learning(12, 13). Despite the existence of mechanisms for evaluating intern performance, no structured feedback system is in place to gather insights from interns as key stakeholders in the program. This gap leaves critical decisions, such as recent structural changes to internship rotations, unsupported by evidence from the perspective of trainees. A recent high-profile legal dispute between the Federation of Uganda Medical Interns and the Ministry of Health highlighted the urgent need for program evaluation mechanisms to ensure that training decisions are informed by stakeholder input(14).

Additionally, concerns over the declining quality of medical graduates, frequent strikes by intern doctors, and reports of suboptimal working conditions underscore the need for systematic evaluation of the internship learning environment(15). To date, the perceptions of intern doctors regarding their clinical learning environment in Uganda remain unexplored.

The theoretical underpinning of this study is Bandura’s Social Cognitive Theory, which emphasizes the dynamic interaction of personal, environmental, and behavioral factors in shaping learning and functioning(16). Key concepts such as self-regulation, modeling, feedback, and self-efficacy are vital in creating an optimal hospital learning environment(17). These principles highlight the importance of clear goals, guided practice, constructive feedback, and reflective opportunities—elements crucial for bridging the gap between theoretical knowledge and practical skills during an internship.

From an educational perspective, the clinical environment significantly influences the learning outcomes of healthcare professionals (18, 19). Recognizing this, the present study sought to evaluate intern doctors' perceptions of their hospital learning environment in Uganda. The findings aim to provide insights that could inform and enhance the structure and delivery of internship training programs in the country.

## METHODS

**Study Design** This was a cross-sectional descriptive study conducted for one month (August 2021) at three internship training centers: Kawempe National Referral Hospital, Kiruddu National Referral Hospital, and Mulago National Referral Hospital (MNRH). The three hospitals were purposively selected based on the fact that they have been at the center of previous intern-related grievances in the form of strikes(20) and host the largest share of trainees per year; almost a quarter of all interns in the whole country. These hospitals collectively host approximately 220 non-residential intern doctors annually for their mandatory training, which follows an apprenticeship model. Of these, Mulago hosts about 160 for all the major 4 major disciplines ( Surgery, Peadiatrics, Internal Medicine, and Obstetrics and Gynecology), Kawempe 40 majoring in obstetrics and Gynecology but also has Peadiatrics and Surgery, and Kiruddu about 45, in all the 4 disciplines but with emphasis on internal medicine. Two hundred (200) medical intern doctors were selected to participate and were given a self-administered Postgraduate Hospital Education Environment Measure (PHEEM) questionnaire. All students responded and returned the questionnaires giving a response rate of 100%.

The study included all medical interns who had been in training at any of the three hospitals for at least one month during their current rotation. Interns with prior training at other hospitals were excluded. Participants were selected using a non-probability consecutive sampling method, meaning they were included as they presented and were accessible to the investigator.

**Data collection tool.**

The PHEEM is an internationally recognized and validated Likert-type instrument designed to assess doctors’ perceptions of their hospital learning environment ((21). The questionnaire takes approximately 15–20 minutes to complete and consists of 40 items related to the learning environment. These items are categorized into three subscales: perception of role autonomy, perception of teaching, and perception of social support.

Responses are indicated on a 5-point Likert scale: 0 (strongly disagree), 1 (disagree), 2 (uncertain), 3 (agree), and 4 (strongly agree). The maximum possible score is 160 (40 items × 4 points), and the minimum is 0, with higher scores indicating a better educational environment.

Four of the 40 items (items 7, 8, 11, and 13) are negatively worded and were scored in reverse. Additionally, three items were modified to better fit the local context. These modifications were informed by a similar study conducted in Nigeria(22). The changes did not affect the psychometric properties of the tool. The modified items are as follows:

**Item 7:**

"There is tribalism in this post" (replacing "There is racism in this post" to reflect the local context).

**Item 11:**

"I am called inappropriately" (instead of "I am bleeped inappropriately" to reflect the local emergency department and call room practices).

**Item 17:**

"My hours of work conform to the civil service rule" (replacing "I work long hours" to align with Ugandan civil service regulations).

**Item 34:**

"The training in this post makes me feel ready to be a Medical Officer/Senior House Officer" (instead of "I feel prepared to be a specialist," reflecting the Ugandan training system).

The tool was pretested with five participants and was deemed suitable for the local context.
**Data Collection Procedure** After obtaining informed consent, participants were given sealed questionnaires to fill out immediately or at their convenience. Completed questionnaires were returned to the unit head within three days.

## Statistical Analysis

**Statistical Assumptions** The PHEEM questionnaire generates ordinal data for each Likert item. When scores are aggregated at the subscale or overall level, near-interval data are obtained, which can be analyzed using parametric methods(23, 24). Though Likert-type data are typically not normally distributed, however the larger sample size (n = 200) allows for the assumption of normality(25).

**Analysis** Cleaned data were imported into an Excel spreadsheet and analyzed using SPSS version 17.0. The four negatively worded items (items 7, 8, 11, and 13) were scored in reverse. Descriptive statistics (frequencies and proportions) were used to describe the baseline characteristics of the participants. Perception scores for the total (sum of all items) and subscales (teaching, autonomy, and social support) were presented as means and standard deviations (SD). Comparisons of perception scores across the four departments—Surgery, Internal Medicine, Pediatrics, and Obstetrics/Gynecology—were made using mean scores ± SD. ANOVA and the Bonferroni test were used to determine statistical significance, with a p-value ≤ 0.05 considered significant.

**Ethical Considerations** Approval for the study was granted by the Department of Pediatrics and Child Health at Makerere University, and by the School of Medicine Research and Ethics Committee (SOMREC) under approval number Mak-SOMREC-2021-107. Administrative clearance was also obtained from the three participating hospitals. All participants provided informed consent before participation in the study. All study procedures were conducted in accordance with the declaration of Helsinki.

## RESULTS

### PARTICIPANT DEMOGRAPHICS

Table 1. Participant demographics.

| VARIABLES | FREQUENCY (N) | PERCENTAGE (%) |
|---|---|---|
| **AGE GROUP** | | |
| 20–24 | 11 | 6.5 |
| 25–29 | 125 | 73.5 |
| 30–34 | 28 | 16.5 |
| 35–39 | 5 | 2.9 |
| 40–44 | 0 | 0 |
| 45–49 | 1 | 0.6 |
| **GENDER** | | |
| Female | 57 | 28.2 |
| Male | 143 | 71.8 |
| **CLINIC ROTATION** | | |
| Accident and Emergency | 1 | 0.6 |
| Internal Medicine | 45 | 26.5 |
| Obstetrics and Gynecology | 32 | 18.8 |
| Pediatrics | 59 | 34.7 |
| SURGERY | 33 | 19.4 |
| **CURRENT INTERNSHIP TRAINING SITE** | | |
| Kiruddu | 30 | 17.6 |
| MNRH | 103 | 60.6 |
| Kawempe | 37 | 21.8 |

A total of 200 intern doctors participated in this study, with 100% of the PHEEM questionnaires being fully completed. The distribution of participants across departments was as follows: 25% (n = 50) from Internal Medicine, 20.5% (n = 45) from Obstetrics and Gynaecology, 30% (n = 60) from Paediatrics, and 24.5% (n = 45) from Surgery. The majority of the participants (72.5%) were aged between 25–29 years, with a male majority (72%). The highest proportion (65%) were undergoing their internship training at Mulago National Referral Hospital, and 34.5% had previously trained at Makerere University.

#### Total PHEEM score

The mean total PHEEM score as represented in Table 1, was **93.27 ± 22.73** (range = 23–143), indicating an educational environment with more positive than negative aspects. This suggests that while trainees perceive certain strengths in their training environment, there are also areas that could benefit from enhancement as may be depicted in the subscale and individual item analysis.

#### SUBSCALE SCORES

**Autonomy**: The mean subscale score for autonomy was **31.35 ± 7.56**, suggesting a generally positive perception of interns' roles in their departments.

**Teaching**: The mean subscale score for teaching was **37.37 ± 11.92**, indicating that the teaching environment is moving in the right direction.

**Social Support**: The mean subscale score for social support was **23.65 ± 6.37**, showing more pros than cons in the support rendered to interns, although the score was relatively low compared to autonomy and teaching.

Table 2
Overall and subscale scores of the PHEEM at the three hospitals

| SCORE | ASSESSMENT | ATTAINED SCORE |
|---|---|---|
| Overall | | 93.27 |
| 0–40 | Very poor | |
| 41–80 | Plenty of problems | |
| 81–120 | A more positive than negative environment | |
| 121–160 | Excellent | |
| **Perception of role autonomy** | | |
| 0–14 | Very poor | 31.35 |
| 15–28 | A negative perception of one's role | |
| 29–42 | A more positive perception of one's role | |
| 43–56 | Excellent perception of one's job | |
| **Perception of teaching** | | |
| 0–15 | Very poor | 37.37 |
| 16–30 | In need of some training | |
| 31–45 | Moving in the right direction | |
| 46–60 | Model teachers | |
| **Perception of social support** | | |
| 0–11 | Non existent | 23.65 |
| 12–22 | Not a pleasant place | |
| 23–33 | More pros than cons | |
| 33–44 | A good supportive environment | |

### Individual item PHEEM score

The lowest recorded score was **1.0** for item 26: "There are adequate catering facilities when I am on call", while the highest was **5.0** for item 16: "I have good collaboration with other doctors in my grade". Thirty-four out of forty questions received a score higher than **2.0**, indicating a generally supportive and suitable educational environment. However, attention should be paid to the questions that scored below **2.0**, which may suggest areas of concern. Specifically, items **9**, **18**, and **32** within the "Autonomy" section, and items **20**, **26**, and **38** within the "Social Support" section had relatively low ratings.

### Comparison of learning environment between different departments

The mean total PHEEM score per department showed that **interns in Internal Medicine** had higher scores compared to all the other departments (p = 0.015), while interns in **Obstetrics and Gynecology** (p = 0.47), **Pediatrics** (p = 0.05) and **Surgery** (p = 1.0) had varying mean scores, but these differences were not statistically significant.

Table 3
Comparison of overall perception by clinic rotation using one-way ANOVA

| Clinic rotation | Mean | Standard Deviation | Frequency | P-value |
|---|---|---|---|---|
| Internal medicine | 97.9 | 24.6 | 49 | 0.015 |
| Obstetrics and Gynecology | 92.6 | 24.5 | 32 | 0.47 |
| Pediatrics | 88.7 | 27.9 | 75 | 0.05 |
| Surgery | 79.9 | 19.8 | 44 | 1.0 |

### Subscale comparison

When comparing the subscale scores for **Autonomy**, **Teaching**, and **Social Support** across departments:

#### Autonomy

Interns in Paediatrics reported higher autonomy than those in other departments especially Surgery (p = 0.007).

#### Teaching

No significant differences in teaching quality were observed among departments (p = 0.052).

#### Social Support

Interns in Internal Medicine rated their social support significantly higher than those in all the other departments (p = 0.019).

Table 4
Comparison of subscale scores across departments

| Domains | Maximum score | Internal medicine (n = 45) | Obs &Gyn (n = 32) | Pediatric (n = 59) | Surgery (n = 34) | ANOVA |
|---|---|---|---|---|---|---|
| Autonomy | 56 | 32.6 ± 7.04 | 30.8 ± 8.3 | 33.1 ± 7.9 | 28.4 ± 6.2 | 0.007 |
| Teaching | 60 | 39.3 ± 13.8 | 37.9 ± 12.6 | 38.3 ± 11.4 | 33.8 ± 9.3 | 0.052 |
| Social support | 44 | 26.4 ± 7.6 | 22.7 ± 6.7 | 23.6 ± 5.7 | 22.4 ± 4.7 | 0.019 |

## DISCUSSION

This study evaluated the perceptions of intern doctors regarding their hospital learning environment using the PHEEM questionnaire at three national referral hospitals in Kampala, Uganda. The training environment for junior doctors in Uganda has often been marred by challenges, including frequent strikes linked to welfare concerns. These strikes, including the one that occurred during our study period, significantly affect the learning experience.

Overall, the intern doctors rated their learning environment positively, with an overall mean PHEEM score of 93.27, indicating a more positive than negative environment (26). This implies that on a global scale, most intern doctors view their learning environment as favorable nonetheless; some areas need attention for improvement. At the subscale level, perceptions were similarly positive, with autonomy scoring 31.35 (suggesting a more positive than negative perception), teaching scoring 37.37 (indicating that the teaching environment is moving in the right direction), and social support scoring 23.65 (more pros than cons). These scores reflect a generally balanced environment, with areas for improvement that could further optimize the learning experience for junior doctors. Similar findings have been reported in other Sub-Saharan African countries and globally, such as Olasoji's study in Nigeria, which reported a higher overall PHEEM score of 98.25 for interns and residents (22).

When comparing learning environments across the three hospitals, we observed a significant difference, with Mulago Hospital scoring the lowest. Well as Mulago Hospital had the greatest majority of participants (60.6%), the difference could equally be attributed to variations in individual PHEEM component scores across hospitals or differences in resources and training practices(27). This is a fundamental observation given the fact that no clear structured and standardized curriculum is followed in internship training; to put it simply, learning occurs through informal apprenticeship. Previous studies have emphasized the positive impact of structured training and assessment in enhancing uniformity, excellent trainee satisfaction, positive feedback, and reduced variability of learning outcomes (28, 29).

The highest scores were observed in areas that emphasized the working relationships between interns, their peers, and supervisors, particularly in items 5, 11, and 16. Positive interactions and collaboration are essential for both medical practice and learning, underscoring the importance of fostering a teamwork-oriented environment for optimal intern development.

The significantly low scores for items 9, 18, and 32 within the autonomy subscale indicate key issues that need addressing. While some interns reported having some form of induction, many lacked access to a junior doctor's handbook, which is an essential tool for orientation. This absence likely contributes to confusion, exploitation, and negligence, which can all hinder both the learning process and the overall well-being of junior doctors. Additionally, the inability to provide continuity of care was another issue highlighted by low scores, linked to inadequate medical supplies and equipment, as frequently raised by doctors' forums. High patient volumes and insufficient staffing, particularly in public facilities, exacerbate these challenges, affecting both intern learning and patient care(30).

The lack of continuity in care limits the internship learning experience, leading to fragmented episodes rather than cohesive, experiential learning (31). While high patient volume could offer valuable exposure, it also contributes to burnout, anxiety, and depression, which can further inhibit reflective learning and the overall quality of training (32). This issue is particularly acute when supervision is lacking, reinforcing the need for structured, supportive environments (33) (34, 35).

Social support, particularly in terms of accommodation, catering, and psychological support, also emerged as a major area of concern, with items 20, 26, and 38 scoring below average. These issues have been reported globally, with similar findings in other low- and middle-income countries. This suggests that the lack of robust personal support for junior doctors may be a universal challenge(22, 36, 37). Addressing these issues is crucial not only for the well-being of the interns but also for improving the overall healthcare system, as resolving these concerns could reduce the time lost to strikes and improve both the learning experience and service delivery. (38). In Uganda, this is fundamental because all strikes by these doctors including the most recent have been centered on the perception of social support during the training.

Interns in the Internal Medicine and Pediatrics departments reported significantly higher overall scores compared to those in Surgery and Obstetrics & Gynecology, with Surgery having the lowest score, which bordered on the category of "plenty of problems." This trend aligns with findings from other studies, such as Ezomike's research on Nigerian urban hospitals. (39). The reasons for these disparities could be related to departmental protocols and priorities, with some departments dedicating more time and resources to intern training than others (22). Moreover, the presence of numerous postgraduate trainees and fellows in the surgical departments may create competition for attention from supervisors, potentially leading to interns feeling neglected. That said, the earlier discussed lack of standard internship training structure perhaps gives room for individual departmental teaching and learning strategies hence the buildup of variations in perceptions of the learning environment. This warrants further investigation through qualitative studies to understand how these dynamics influence intern perceptions.

At the subscale level, surgery scored the lowest in both autonomy and teaching quality. These findings underscore the importance of recognizing departmental-specific challenges and the need for tailored improvements (39). It also highlights that learning environments can be locally controlled and effectively monitored within departments to ensure they meet acceptable standards hence improving the quality of training for interns (40).

The reliability of the PHEEM tool was confirmed by a Cronbach's alpha value of 0.9193, demonstrating high internal consistency. This result is consistent with previous studies in Africa and globally, which have affirmed the reliability of the PHEEM in assessing the quality of the hospital learning environment (26, 41, 42). This study represents the first documented validation of the PHEEM in Uganda, which is significant because it suggests that the tool is applicable in this context and can be used for ongoing monitoring of junior doctors' learning environments in Uganda.

The findings of this study highlight key insights into the internship training environment in Uganda. Notably, the learning environment varies significantly across departments and hospitals. This underscores the need for regular assessments, using tools like PHEEM, to monitor and improve the learning environment on an ongoing basis. The PHEEM, with its proven reliability in the Ugandan context, can be an effective instrument for driving improvements and ensuring the quality of training.

### STUDY LIMITATIONS AND STRENGTHS

Several limitations were considered in this study. The data collection was interrupted by an intern doctor's strike regarding poor welfare and the absence of medical consumables. This may have introduced a bias toward negative perceptions of the learning environment. Additionally, the COVID-19 pandemic with its prioritization of emergency patients and the overwhelming patient numbers may have altered hospital operations, potentially influencing interns' perceptions of their training. The new internship rotation cycle, which includes major (6 months) and minor (3 weeks) rotations, might also have contributed to dissatisfaction among interns, affecting their overall perception of the learning environment. It may call for a qualitative inquiry to explain why interns rated the learning environment as positive despite the above limitations. It is also important to note that our nonprobability sampling technique could have introduced selection bias, but this is negligible since our sample size was 90.9% of the study population. The other limitation is that the PHEEM tool lacks gradual transition in mean scores for characterization of results and so may not give the exact difference between two close levels. This was however mitigated by doing a detailed subscale analysis to give an adjusted view of the overall picture.

However, our study addresses the knowledge gap in the East African region and benefits from a large sample size of 200 participants. Its multi-center nature gathers data from multiple locations making it rich although the findings would have been more generalizable if different study settings like rural, and private not for profit sites were included. This study provides a comprehensive assessment of the internship-learning environment in Uganda, which can inform future interventions and policy decisions.

## CONCLUSIONS

The hospital learning environment in the three hospitals assessed is generally more positive than negative, with room for improvement. There are weaknesses particularly evident in social support and role autonomy. Differences in perception of the learning environment exist across departments with medical departments: Internal Medicine and Pediatrics scoring better than the surgical departments of: Surgery and Obstetrics and Gynecology.

## RECOMMENDATIONS

The national internship council should gear towards structuring and standardization of the internship training including the development of a uniform curriculum including standard assessment.

Attention should be focused on improving existing social welfare concerns. Hospitals and departments should regularly monitor their learning environment using tools like the PHEEM tool to ensure the maintenance of quality standards.

University teaching hospitals may need to devise ways of balancing intern supervisory responsibilities with the rest of the students.

**SUGGESTIONS FOR FURTHER STUDY.**

Future studies could extend this research to include private and private-not-for-profit hospitals for comparison. Investigating the internship-learning environment at rural training sites, and comparing these with urban hospitals, would also provide valuable insights into strategies to retain doctors in rural communities. Further qualitative research is needed to explore the specific causes of inter-departmental differences in the learning environment, to identify targeted solutions, and to get a detailed understanding of how these factors shape the perception of the learning environment.

## Declarations

ETHICAL APPROVAL: Ethical approval was sought and granted by Makerere University College of Health Sciences, School of Medicine institutional review board under the number:

**Mak-SOMREC-2021-107**

All participants provided informed consent before taking part in the study. All study procedures were conducted in accordance with the Helsinki declaration.

CONSENT FOR PUBLICATION: Not applicable.

AVAILABILITY OF DATA AND MATERIALS: The data set for this article is available as an attached file in this published article.

**COMPETING INTERESTS: the authors declare that they have no competing interests.**

**FUNDING: This research was supported by the Fogarty International Center of the National Institutes of Health under Award Number 1R25TW011213. The content is solely the responsibility of the authors and does not necessarily represent the official views of the National Institutes of Health.**

**AUTHORS CONTRIBUTIONS**

**M. K Developed the research proposal and spearheaded data collection and writing of the final book.**

**A.G.M Faculty supervisor offered corrections and guidance from time to time.**

**I.M. Postgraduate supervisor guided the research study from concept development to the final manuscript.**

**S.K. The research supervisor offered direction concerning every step of the research study.**

**C.M. Analyzed the data and participated in the writing of the final book.**

**M.P. Participated in proposal and manuscript writing.**

**ACKNOWLEDGEMENTS: Not applicable.**
